# Rezidivierende SARS-CoV-2-Infektionen bei Immundefizienz

**DOI:** 10.1007/s00108-022-01424-7

**Published:** 2022-10-28

**Authors:** L. Tometten, J. J. Malin, E. Pracht, P. J. Bröckelmann, C. Horn, R. Sprute, C. A. Langhorst, M. Hallek, G. Fätkenheuer, J. Rybniker

**Affiliations:** 1 Klinik I für Innere Medizin, Universität zu Köln, Medizinische Fakultät und Uniklinik Köln, Kerpener Str. 62, 50937 Köln, Deutschland; 2 Medizinische Fakultät und Uniklinik Köln, Klinik für Dermatologie und Venerologie, Universität zu Köln, Köln, Deutschland

**Keywords:** Immundefizienz, B‑Zell-Neoplasie, Monoklonale Antikörper, COVID-19, Antivirale Therapie, Immunodeficiency, B‑cell neoplasia, Monoclonal antibodies, COVID-19, Antiviral treatment

## Abstract

Ein Patient mit Immundefizienz im Rahmen eines B‑Zell-Lymphoms wurde seit Beginn der SARS-CoV-2-Pandemie wiederholt positiv auf SARS-CoV‑2 getestet und zweimal stationär versorgt. Chronische und rezidivierende SARS-CoV-2-Infektionen gefährden die Gesundheit von Patientinnen und Patienten mit Immundefizienz. Insbesondere aufgrund neuer Virusvarianten mit Immune-escape-Mechanismen sind die Therapieoptionen eingeschränkt. Die Versorgung immundefizienter Patienten mit SARS-CoV-2-Infektion stellt behandelnde Ärztinnen und Ärzte in der aktuellen Pandemie vor große Herausforderungen.

## Abstract (en)

A patient with immunodeficiency due to a B-cell lymphoma has repeatedly been tested positive for SARS-CoV‑2 during the ongoing SARS-CoV‑2 pandemic and has twice received in-hospital treatment. Chronic and recurrent SARS-CoV‑2 infections are a threat to the individual health of immunodeficient patients. Only few therapeutic options are available especially due to emerging virus variants with immune escape mechanisms. The medical care of immunodeficient patients with SARS-CoV‑2 infections is a great challenge to the treating physician in the ongoing pandemic.

## Anamnese

Ein Mitte 60-jähriger Patient wurde Ende Februar 2022 mit Nachweis einer Severe-acute-respiratory-syndrome-virus-2(SARS-CoV-2)-Infektion mit Fieber und Husten stationär aufgenommen. Anamnestisch hatte der Patient bereits im Dezember 2020, im Juli und September 2021 sowie Anfang Februar 2022 SARS-CoV-2-Infektionen durchgemacht.

Der Patient litt an einem 2018 diagnostizierten B‑Zell-Non-Hodgkin-Lymphom (follikuläres Lymphom) und erhielt bis Juli 2021 eine Erhaltungstherapie mit Rituximab. Bei Progress des Lymphoms wurde die Therapie im August 2021 auf Obinutuzumab/Bendamustin umgestellt. Es musste somit von einer ausgeprägten erkrankungs- und therapieassoziierten Immundefizienz ausgegangen werden.

## Befund

**Figure 1:**
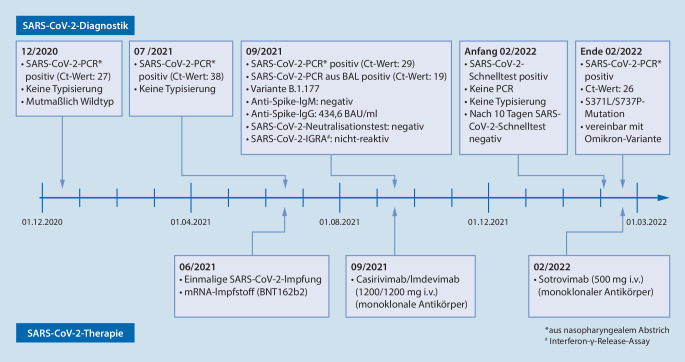

Der Patient stellt sich zwischen Dezember 2020 und März 2022 mehrfach mit leichten und mittelschweren Symptomen einer SARS-CoV-2-Infektion vor. Virologische Befunde sind in Abb. 1 zusammengefasst. Symptome wie Fieber, allgemeine Erschöpfung sowie Husten stehen im Vordergrund. Eine ausgeprägte Dyspnoe liegt zu keinem Zeitpunkt vor. Der auskultatorische Befund ist größtenteils unauffällig.

## Diagnose

- Rezidivierende SARS-CoV-2-Infektionen bei Immundefizienz

## Therapie und Verlauf

Im Dezember 2020 wurde erstmalig eine SARS-CoV-2-Infektion mittels PCR aus einem nasopharyngealen Abstrich nachgewiesen (Abb. 1). Eine Virustypisierung erfolgte nicht. Mutmaßlich handelte es sich um eine Infektion mit dem Wuhan-Typ, der zu diesem Zeitpunkt in Deutschland vorherrschte. Der Patient blieb beschwerdefrei und die Betreuung erfolgte ambulant.

Im Juni 2021 erfolgte eine einmalige Impfung gegen SARS-CoV‑2 mit dem mRNA-Impfstoff BNT162b.

Im Juli 2021 traten Erkältungssymptome auf. Der nasopharyngeale PCR-Abstrich auf SARS-CoV‑2 war positiv. Eine Typisierung war aufgrund der niedrigen Viruslast im Probenmaterial nicht möglich. Weitere Abstriche erfolgten nicht, sodass unklar ist, ob zu diesem Zeitpunkt eine erneute Infektion oder lediglich der Nachweis persistierenden Virusmaterials sowie eine andere respiratorische Infektion vorlag. Die Versorgung erfolgte erneut ambulant.

**Figure 2:**
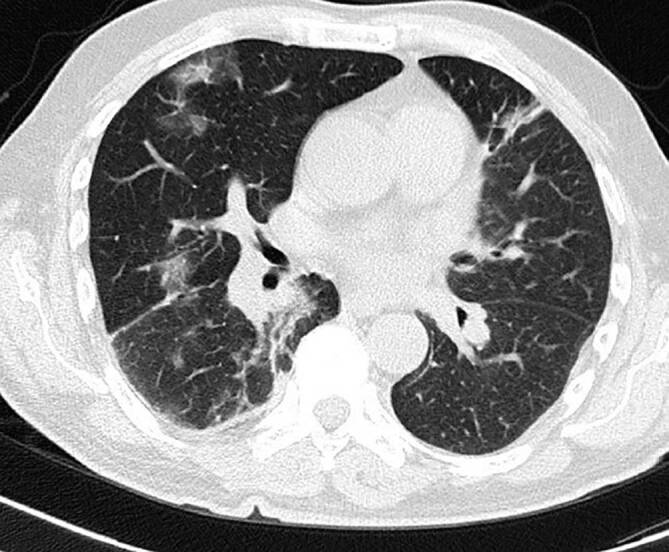

Aufgrund der im August eingeleiteten Therapie mit Obinutuzumab und Bendamustin war der Patient im September 2021 neutropen und entwickelte Fieber und Husten. Stationär wurde erneut eine SARS-CoV-2-Infektion diagnostiziert. Aufgrund des Fiebers in Neutropenie erfolgten bei respiratorischer Symptomatik eine Computertomographie der Lunge sowie eine bronchoalveoläre Lavage zum Ausschluss opportunistischer Erreger. In der Computertomographie zeigten sich zu einer COVID-19-Erkrankung passende Infiltrate beidseits (Abb. 2).

Die bronchoalveoläre Lavage ergab ebenfalls einen SARS-CoV-2-Nachweis. Weitere Erreger wurden nicht gefunden. In der serologischen Diagnostik war das Anti-Spike-IgG positiv. Das Anti-Spike-IgM und ein Antikörperneutralisationstest waren jedoch negativ, der SARS-CoV-2-Interferon-γ-Release-Test zur Abschätzung einer T‑Zell-Antwort war nicht reaktiv. Eine antibakterielle Therapie mit Piperacillin/Tazobactam wurde bei fehlendem Hinweis auf eine bakterielle Infektion beendet.

Aufgrund des Risikos für einen schweren Verlauf unter Chemoimmuntherapie erfolgte die einmalige Gabe der rekombinanten Antikörperkombination Casirivimab/Imdevimab (1200/1200 mg). Der Patient entfieberte rasch, die Abstriche auf SARS-CoV‑2 waren im Verlauf negativ. Durch Sequenzierung der Probe wurde nachträglich eine Infektion mit der Variante B.1.177 nachgewiesen.

Anfang Februar 2022 kam es erneut zu Erkältungssymptomen. Ein Schnelltest auf SARS-CoV‑2 war positiv. Eine PCR-Diagnostik und eine ärztliche Vorstellung erfolgten nicht. Der SARS-CoV-2-Schnelltest war am Ende der häuslichen Isolierung negativ.

Ende Februar 2022 traten wieder Fieber und Husten auf. Die SARS-CoV-2-PCR war positiv. Die Mutationsanalyse zeigte eine S371L/S373P-Mutation, vereinbar mit der Omikron-Variante. Stationär wurde bei hohem Risiko für einen schweren Verlauf der monoklonale Antikörper Sotrovimab (500 mg) verabreicht. Der Patient konnte rasch entlassen werden und war zuletzt im Juli 2022 ohne residuelle Beschwerden.

## Diskussion

Der vorgestellte Fall zeigt eindrücklich die Komplexität der Krankheitsverläufe von SARS-CoV-2-Infektionen bei Patientinnen und Patienten mit Immundefizienz. Rezidivierende Infektionen und die Entstehung von Immune-escape-Varianten gefährden insbesondere Patientinnen und Patienten mit Immundefizienz und stellen die behandelnden Ärztinnen und Ärzte vor große Herausforderungen. Prinzipiell sind unterschiedliche prophylaktische und therapeutische Optionen gegen SARS-CoV‑2 verfügbar. Jedoch ist die Mehrzahl der zulassungsrelevanten Studien bei ungeimpften Patientinnen und Patienten und bei Patientinnen und Patienten ohne Immundefizienz durchgeführt worden, sodass evidenzbasierte Therapieentscheidungen erschwert sind.

### Impfungen

Impfungen gegen SARS-CoV‑2 haben zu einer effektiven Senkung der SARS-CoV-2-assoziierten Morbidität und Mortalität geführt. Bei Immundefizienz ist jedoch das Risiko einer eingeschränkten Impfantwort deutlich erhöht. Personen mit hämatoonkologischen Erkrankungen und nach Organtransplantation erreichen nach aktiver Immunisierung deutlich seltener eine Serokonversion als Immunkompetente [3, 8], wobei die Bedeutung der Serokonversion für den Schutz vor schweren Krankheitsverläufen nicht abschließend geklärt ist [7]. Anzunehmen sind zusätzliche protektive Effekte durch die zelluläre Immunantwort, welche auch bei anhaltend seronegativen Personen durch die Impfung hervorgerufen werden können. Die Ständige Impfkommission (STIKO) empfiehlt bei Patientinnen und Patienten mit Immundefizienz daher intensivierte Impfschemata. Allgemein entspricht eine durchgemachte Infektion in den Impfschemata der STIKO einer Impfdosis. In dem vorliegenden Fall ist eine Impfung erfolgt, aufgrund wiederholter SARS-CoV-2-Infektionen und der Gabe von monoklonalen Antikörpern wurden jedoch keine weiteren Impfungen durchgeführt. Nach aktuellen Empfehlungen spricht die Gabe von monoklonalen Antikörpern nicht mehr gegen die Durchführung einer Impfung. Die Grundimmunisierung bei Personen mit relevanter Einschränkung der Impfantwort sollte aus mindestens drei Impfdosen bestehen, zusätzlich sind zwei Auffrischungsimpfungen im Abstand von mindestens drei Monaten empfohlen. Der zeitliche Abstand der Verabreichung einer Impfdosis nach akuter Infektion sollte mindestens vier Wochen im Rahmen der Grundimmunisierung und drei Monate im Rahmen der Auffrischung betragen. Die gewählte Gesamtanzahl an Impfungen nach durchgemachten SARS-CoV-2-Infektionen ist jedoch nicht strikt festgelegt und eine Einzelfallentscheidung. Serologische Diagnostik kann bei der Festlegung der Anzahl an Impfstoffdosen unterstützend erfolgen [9].

### Monoklonale Antikörper

Gegen das SARS-CoV-2-Spike-Protein gerichtete monoklonale Antikörper können einen wichtigen Beitrag in der COVID-19-Frühtherapie leisten und zu einer Reduktion von Hospitalisierungen und Mortalität führen, wenn die jeweilige Virusvariante im Wirkspektrum der eingesetzten Substanz ist [4]. In kleineren Fallstudien zeigte diese Therapieform ein gutes klinisches und virologisches Ansprechen bei Patientinnen und Patienten mit B‑Zell-Neoplasien [5]. Eine Herausforderung bei der Anwendung monoklonaler Antikörper ist das Auftreten neuer Virusvarianten mit Immune-escape-Mechanismen. Klinische Daten zur Effektivität der monoklonalen Antikörper liegen für die aktuell vorherrschende Variante meist nicht vor. Die Wirksamkeit muss daher von *In-vitro*-Untersuchungen abgeleitet werden. Der Einsatz eines monoklonalen Antikörpers sollte dann in Abhängigkeit von der vorherrschenden Variante erfolgen. Die Wirksamkeit der in dem vorliegenden Fall genutzten Antikörper Casirivimab/Imdevimab und Sotrovimab gegenüber den derzeit zirkulierenden Omikron-Varianten BA.2 und BA.5 ist *in vitro* stark beeinträchtigt. Gerade bei immundefizienten Patientinnen und Patienten kann eine Virustypisierung erfolgen, da hier protrahierte Infektionsverläufe mit Varianten vorliegen können, für die noch wirksame Antikörpertherapien verfügbar sind. Das intramuskulär zu verabreichende Kombinationspräparat Tixagevimab/Cilgavimab zeigt *in vitro* eine neutralisierende Wirkung gegenüber den BA.2- und BA.5-Subvarianten und kann bei Personen mit Immundefizienz, bei denen ein Ausbleiben der schützenden Immunantwort nach Impfung zu erwarten ist, prophylaktisch im Rahmen einer passiven Immunisierung verabreicht werden [1]. Die Verabreichung ist auch vor Beendigung der vorgesehenen Impfserie möglich. Neben der prophylaktischen Anwendung wurde die Indikation für Tixagevimab/Cilgavimab vor kurzem erweitert. Eine therapeutische Anwendung von Tixagevimab/Imdevimab im Rahmen einer akuten SARS-CoV-2-Infektion ohne Sauerstoffpflichtigkeit bei erhöhtem Risiko für einen schweren Krankheitsverlauf kann demnach ebenfalls erwogen werden.

### Antivirale Substanzen

Neben den monoklonalen Antikörpern stehen für die Behandlung einer SARS-CoV-2-Infektion Medikamente mit unterschiedlichen antiviralen Wirkmechanismen zur Verfügung. Diese Substanzen wirken im Gegensatz zu den monoklonalen Antikörpern bei allen derzeit zirkulierenden Virusvarianten. Die Anwendung der in Tablettenform verfügbaren Substanzen Molnupiravir (Lagevrio) und Nirmatrelvir/Ritonavir (Paxlovid) ist für Personen mit Risikofaktoren (insb. bei Alter ≥ 65 Jahre und/oder inkomplettem Impfschutz) in den ersten 5 Tagen nach Symptombeginn empfohlen [2]. Daten aus randomisiert-kontrollierten Studien zur Anwendung bei immundefizienten Patientinnen und Patienten lagen zum Zeitpunkt der Einreichung des Artikels noch nicht vor. Mit Remdesivir gibt es eine weitere antivirale Substanz, die jedoch ausschließlich intravenös verabreicht werden kann. Patientinnen und Patienten mit Immundefizienz werden häufig von klinischen Studien ausgeschlossen, dennoch deuten theoretische Erwägungen und klinische Beobachtungen auf einen Nutzen von antiviralen Therapien bei diesen Patientinnen und Patienten hin. Bei erhöhtem Risiko für einen schweren Krankheitsverlauf, insbesondere bei Immundefizienz mit Risiko für unzureichendes Impfansprechen, sollte die Gabe antiviraler Substanzen in der Frühphase der Infektion erfolgen [10]. Der klinische Nutzen antiviraler Therapien bei Patientinnen und Patienten mit Immundefizienz besteht wahrscheinlich auch bei der Verabreichung in späteren Erkrankungsphasen [6].

Zusammengefasst stellt das Management von Personen mit Immundefizienz in der COVID-19-Pandemie behandelnde Ärztinnen und Ärzte vor anhaltende Herausforderungen. Für die adäquate Versorgung müssen Therapieoptionen unter Berücksichtigung der aktuellen epidemiologischen Lage permanent diskutiert und angepasst werden. Die rasche Entwicklung von Immune-escape-Varianten erfordert bei der Gabe von therapeutischen Antikörpern eine individualisierte Herangehensweise, und die medikamentöse Therapie muss aufgrund mangelnder Studien und Zulassungen oftmals „off label“ erfolgen.

## Fazit für die Praxis

- Ein vollständiger Impfstatus gegen SARS-CoV‑2 ist bei Patientinnen und Patienten mit Immundefizienz essenziell, auch wenn ein ausreichender Impfschutz häufig schwer zu erzielen ist.
- Die therapeutische Anwendung von monoklonalen Antikörpern muss abhängig von zirkulierenden Virusvarianten ausgewählt werden. Bei Immundefizienz kann eine Virustypisierung zur Selektion passender therapeutischer Antikörper beitragen.
- Die Anwendung der antiviralen Medikamente erfolgt unabhängig von der zugrunde liegenden Variante.
